# Assessing Relations between PTSD’s Dysphoria and Reexperiencing Factors and Dimensions of Rumination

**DOI:** 10.1371/journal.pone.0118435

**Published:** 2015-03-04

**Authors:** Meredith A. Claycomb, Li Wang, Carla Sharp, Kendra C. Ractliffe, Jon D. Elhai

**Affiliations:** 1 Department of Psychology, University of Toledo, Toledo, Ohio, United States of America; 2 Key Laboratory of Mental Health, Institute of Psychology, Chinese Academy of Sciences, Beijing, China; 3 Department of Psychology, University of Houston, Baylor College of Medicine, The Menninger Clinic, Houston, Texas, United States of America; 4 San Francisco Veteran’s Affairs Medical Center, San Francisco, California, United States of America; 5 Department of Psychiatry, University of Toledo, Toledo, Ohio, United States of America; Central Institute of Mental Health, GERMANY

## Abstract

The purpose of the present study was to investigate the relations between posttraumatic stress disorder’s (PTSD) dysphoria and reexperiencing factors and underlying dimensions of rumination. 304 trauma-exposed primary care patients were administered the Stressful Life Events Screening Questionnaire, PTSD Symptom Scale based on their worst traumatic event, and Ruminative Thought Style Questionnaire (RTSQ). Confirmatory factor analyses (CFAs) were conducted to determine the dysphoria and reexperiencing factors’ relationships with the four factors of rumination. Results revealed that both the dysphoria and reexperiencing factors related more to problem-focused thinking and anticipatory thoughts than counterfactual thinking. Additionally, the reexperiencing factor related more to anticipatory thinking than repetitive thinking. Clinical and theoretical implications are discussed.

## Introduction

Recent research has examined cognitive mechanisms and their relations to posttraumatic stress disorder (PTSD) [1]. Rumination, the tendency to perseverate on negative emotions or events, is one such mechanism. Little is known about the specific aspects of rumination that play a role in PTSD development. Therefore, the intent of this paper is to investigate the relationship between several dimensions of PTSD and rumination. In order to comprehensively examine this relationship, PTSD and rumination were compared at the latent factor-level.

### Rumination and PTSD

Rumination can be defined as intrusive and repetitive negative thinking about past experiences and/or emotions, and is relatively stable over time [2]. Ehlers and Clark’s cognitive model of PTSD [3, 4] posited that negative interpretations of the traumatic memory results in heightened levels of distress, which in turn leads to such maladaptive cognitive strategies as rumination. In the context of this model, rumination has been deemed a possible cognitive avoidance strategy [5], in which the person engages in rumination about the meaning, causes, and consequences of the trauma, but not the actual events of the trauma [6]. This form of maladaptive coping [4] results in faulty processing and increased symptom expression (e.g. negative affect, reexperiencing) [7]. Rumination has been found to longitudinally predict PTSD symptoms up to 6 months following a traumatic event [8]. Michael, Halligan, Clark and Ehlers [9] found that assault survivors with PTSD reported significantly more rumination overall, and more time spent ruminating than assault survivors without PTSD. Furthermore, several types of rumination appear to affect PTSD severity, such as ineffective thinking, perseveration over why something happened and what would have happened had circumstances been different, and continued pressure to ruminate [9].

### Rumination’s Underlying Structure

Several measures exist that define rumination from different theoretical perspectives and appear to measure different types of ruminative experiences. The more widely used rumination questionnaires measure ruminative thinking about depression symptoms, rumination on sadness and circumstances surrounding it, distress about intrusive thoughts following a distressing event, and searching for meaning in negative experiences [10]. These different theoretical approaches to measuring rumination can provide challenges to understanding its mechanisms and relations with other disorders, particularly regarding rumination’s factor structure.

One newer instrument for measuring rumination is the Ruminative Thought Style Questionnaire (RTSQ). This instrument was initially developed to measure a general style of ruminative thinking that was not subject to bias by valence, item content, and time-specific information [11], which is a limitation seen in other rumination measures. Another limitation the RTSQ appears to overcome is its focus on general style of ruminative thinking, and not just depressive content [12]. This makes the RTSQ a promising global measure of rumination and worthy of further investigation, particularly when it comes to assessing rumination’s relationships with other disorders that do not necessarily fall under the umbrella of the depressive disorders.

Tanner, Voon, Hasking and Martin [12] used a large adolescent sample to investigate the underlying symptom dimensions of rumination measured by the Ruminative Thought Style Questionnaire [12]. They conducted an exploratory factor analysis (*N* = 1181), then a confirmatory factor analysis (*N* = 1181), and found that a four-factor structure of rumination fit best, supporting rumination as a multi-dimensional construct [12]. The four factors were labeled as follows: problem-focused thoughts, counterfactual thinking, repetitive thoughts, and anticipatory thoughts [12].

The first factor of rumination, problem-focused thoughts (R-PFT), can be conceptualized as deficits in problem-solving ability and information processing, and was found to predict increased levels of distress in the Tanner, Voon, Hasking and Martin [12] adolescent sample. The second factor, counterfactual thinking (R-CT), is defined as imagining and perseverating on imagined alternative outcomes to various situations that will happen and/or have all ready happened, and is linked to emotions like regret, disappointment, shame, and guilt [12]. The third factor is repetitive thoughts (R-RT) and focuses on the frequency and repetitiveness of cognitions, with its most defining features being the persistence, intrusiveness, and involuntary nature of the thoughts. This factor also increased levels of distress in the Tanner, Voon, Hasking and Martin [12] sample. Lastly, the fourth factor, anticipatory thoughts (R-AT), is indicative of intrusive and persistent perseveration over future possible events [12].

### PTSD’s Underlying Structure

We investigated rumination’s relations with latent factors of PTSD. Early PTSD factor analytic research has focused on the *DSM-IV* three-factor model [13]. In a literature review by Elhai and Palmieri [13], it was determined that there were two four-factor models that fit the symptom structure of PTSD: the emotional numbing model [14] and the dysphoria model [15].

King, Leskin, King and Weathers [14] proposed an emotional numbing model of PTSD that consists of four factors: reexperiencing (PTSD-RE), avoidance (PTSD-AV), numbing (PTSD-N), and hyperarousal (PTSD-HYP). This model differs from three-factor models because it divides numbing and avoidance into two distinct factors [14] (see Table 1). The dysphoria model of PTSD (using *DSM-IV* criteria) has four factors consisting of reexperiencing, avoidance, dysphoria (PTSD-DYS), and hyperarousal symptoms [15]. In this model, the larger PTSD-DYS factor has eight symptoms from the *DSM-IV* numbing and arousal factors that load onto it [15] (see Table 1).

**Table 1:** Item Mapping for PTSD Models.

Items	DSM-IV	Dysphoria	Emotional Numbing	5-Factor Model
Intrusive thoughts	R	R	R	R
Nightmares	R	R	R	R
Reliving trauma	R	R	R	R
Emotional cue reactivity	R	R	R	R
Physiological cue reactivity	R	R	R	R
Avoidance of thoughts	A, N	A	A	A
Avoidance of reminder	A, N	A	A	A
Trauma-related amnesia	A, N	D	N	D
Loss of interest	A, N	D	N	D
Feeling detached	A, N	D	N	D
Feeling numb	A, N	D	N	D
Hopelessness	A, N	D	N	D
Difficulty sleeping	H	D	H	DA
Irritability/anger	H	D	H	DA
Difficulty concentrating	H	D	H	DA
Hypervigilance	H	H	H	AA
Easily startled	H	H	H	AA

*Note*: R = Reexperiencing, A = Avoidance, N = Numbing, H = Hyperarousal, D = Dysphoria, DA = Dysphoric Arousal, AA = Anxious Arousal

There has been debate as to which model is the better fit for PTSD. Yufik and Simms [16] conducted a meta-analysis in which they looked at 40 covariance matrices. These had different PTSD assessments (based on *DSM-IV*) and different trauma samples. Their analysis revealed the important findings that although the four-factor models have the best fit for PTSD, the dysphoria model had a consistently better fit than the emotional numbing model and this was not affected by the measure used or the type of trauma sample [16].

Another PTSD model that has received strong support in the literature is the five-factor dysphoric arousal model [17]. In this model, the PTSD-RE factor is made up of the same five symptoms as the other factor models of PTSD, and the PTSD-AV and PTSD-HYP (renamed anxious arousal in the five-factor model) are unchanged from the emotional numbing model [17]. The biggest change is that the PTSD-DYS factor is split into two factors, a numbing factor and a dysphoric arousal factor [17] (see Table 1 for item mapping).

To date, these are the PTSD factor models that have received the most support in the literature, with the majority of studies focusing on the four-factor emotional numbing and dysphoria models.

### Rumination and PTSD Factors

We focus here on the dysphoria model in relation to dimensions of rumination. In comparison to the numbing model, the dysphoria model has been documented with having a more consistent, better fit across different trauma samples and using different assessment types [16]. According to Yufik and Simms [16] meta-analysis, the dysphoria model [15] fits these requirements over the emotional numbing model [14]. There is evidence in the existing literature for the relatively new five-factor dysphoric arousal model [17]. However, because no previous study has examined PTSD’s factor structure in relation to rumination, we believe it is best to start this line of inquiry with the four-factor dysphoria model rather than the newer five-factor model.

In studying rumination’s relations to PTSD dimensions, we focus specifically on two specific PTSD factors: dysphoria and reexperiencing. There are several reasons we choose to initially focus on these two factors. Specific aspects of rumination could have distinctive relations to the PTSD-RE factor. Several studies have shown that people engage in cognitive strategies such as rumination in order to control the intrusive images, part of the PTSD-RE factor [5, 18]; however, there are no studies to date that have explored this research question.

We also focus on the PTSD-DYS factor for several reasons. Some of the symptoms from the PTSD-DYS factor overlap with other mood and anxiety disorders (e.g., sleep, irritability, concentration problems, hopelessness, loss of interest). Forbes and colleagues [19] found that PTSD-HYP, PTSD-RE, and PTSD-AV factors were more related to Fear disorders (e.g., disorders like simple phobia, panic disorder, etc) and that the PTSD-DYS cluster of symptoms was more related to disorders falling under the Anxious-Misery umbrella (e.g., generalized anxiety disorder and depression). Additionally, a recent study by Gros, Simms and Acierno [20] suggested that the PTSD-DYS factor was more related to depression than other PTSD factors, which could account for PTSD’s high comorbidity with depression. In addition to dysphoria, PTSD and depression also share many common cognitive processes, including rumination. In order to better understand this comorbid relationship, it makes sense to study common symptom clusters (e.g., dysphoria) and common cognitive processes (e.g., rumination) to determine if their co-relations play a role in PTSD development and maintenance. Because both rumination and the PTSD-DYS factor are associated with PTSD, depression, and general distress, it would be expected that dysphoria and rumination could be related to each other; however this research question has also not been explicitly tested.

### Aims

Because of the limited research on the relationship between cognitive mechanisms, particularly rumination, and PTSD’s factor structure, it is important to investigate this area for its treatment implications. This is especially important given rumination’s connections to PTSD symptom maintenance and to PTSD and distress. Therefore, we specifically investigated the relations between rumination’s dimensions and the PTSD-DYS and the PTSD-RE factor. Doing so at the level of latent variables, rather than observed variables, ensures maximum precision in estimating the strength between variables.

Due to the exploratory nature of this study and the novelty of using the RTSQ as a measure of rumination’s factor structure, we put forth the following hypothesis based predominantly on theory: R-PFT is defined by Tanner, Voon, Hasking and Martin [12] as deficits in information processing. This falls in line with Ehlers and Clark’s [3] theory of rumination’s role in PTSD maintenance, in which people have negative appraisals of the traumatic event, resulting in heightened symptom levels, and maladaptive coping strategies such as rumination that do not allow sufficient processing of the negative event, resulting in even higher and prolonged symptom levels (e.g., distress, more intrusive thoughts). We therefore hypothesize that R-PFT will be most related to PTSD-RE and PTSD-DYS over other rumination factors.

## Method

### Participants and Procedure

The data utilized in this study were part of a larger dataset originally featured in an article on PTSD’s structure by Elhai, Naifeh, Forbes, Ractliffe and Tamburrino [21]. The University of South Dakota’s Institutional Review Board approved this study. Data collection for this study occurred in 2010 in a public, primary care clinic located in a medium-sized U.S. Midwestern city. This clinic was affiliated with the state university medical school. Adults presenting to the clinic between the ages of 18 to 65 were recruited to participate in a study and were informed they would not receive any compensation. The study utilized an informed consent statement that did not require the participants’ signature and was anonymous. Participants read the consent statement and if they agreed to participate, were given the survey. This was part of a waiver of signed consent granted by the University of South Dakota’s Institutional Review Board. The survey was completely anonymous and did not ask for identifying information.

In total, 551 adults were recruited for study participation. Of those, 52 were excluded due to either not speaking English or not being affiliated with the clinic. Out of the remaining 499 potential participants, 411 agreed to participate (82% response rate).

### Measures

The following demographic characteristics were collected: age, gender, years of education, race, marital status, employment status, and household income.

The *Stressful Life Events Screening Questionnaire* (SLESQ) [22] is a self-report questionnaire that assesses 12 *DSM-IV* PTSD Criterion A traumatic events, as well as an additional “catch-all” item. Questions were only asked about the traumas, with no follow-up questions about aspects of the trauma. Participants were asked to specify the trauma they found most distressing. This questionnaire has demonstrated adequate test-retest reliability (κ = .73) and convergent validity [22].

The *PTSD Symptom Scale* (PSS) [23] is a 17-item self-report questionnaire that assesses for PTSD symptoms based on *DSM-IV* criteria. Symptom severity over the past 2 weeks is rated on a 4-point Likert scale ranging from 0 (“not at all”) to 3 (“5 or more times per week/very much/almost always”), with higher scores indicating more severity. PTSD symptoms were rated based on the most-distressing traumatic event reported by the participant on the SLESQ. The questionnaire has good overall psychometrics, including good test-retest reliability (κ = .74), internal consistency of. 91 (.94 in present sample), and convergent validity [23]. Although the symptoms are self-reported, probable PTSD diagnosis was based on *DSM-IV* criteria and symptoms were considered endorsed if the participant scored a 1 (“once per week or less/a little bit/once in awhile”) or higher on an item.

The *Ruminative Thought Style Questionnaire* (RTSQ) [11] is a self-report questionnaire that assesses for different types of ruminative thinking. It includes 20 items that are rated on a 7-point Likert scale ranging from 1 (“does not describe me at all”) to 7 (“describes me very well”). This measure has demonstrated adequate psychometrics, including the following: internal consistency (Cronbach’s α = .95; .94 in present sample) [11], and convergent validity with the widely used Response Style Questionnaire (RSQ), Global Rumination Scale (GRS), and the Beck-Depression Inventory-II [11]. Additionally, it appears to be a more global measure of ruminative thinking because it measures the overall general tendency to think repetitively, recurrently and intrusively [11] as compared to the RSQ, which focuses on rumination over depressive content [12]. Drawing on a sample of college students, Brinker and Dozois [11] proposed and validated a unitary construct of rumination, but a recent factor analytic study yielded a better-fitting four-factor solution with 15 of the items [12]. The four factors were labeled as the following: problem-focused thoughts, counterfactual thinking, repetitive thoughts, and anticipatory thoughts [12].

### Exclusions and Missing Data

Two participants did not complete items on the SLESQ, leaving 409 participants that completed the questionnaire. Of these participants, 329 endorsed at least one traumatic event. Among the 329 trauma-exposed participants, 19 did not answer items on the PSS, bringing the sample size to 310. Six participants were excluded from additional analyses due to not completing over 50% of items on the RTSQ and/or PSS, resulting in a final sample size of 304. CFA analyses were conducted with Mplus 7.1 software [24]. Missing values were estimated using maximum likelihood procedures with a pairwise present approach.

### Analysis

Three Confirmatory Factor Analyses (CFAs) were conducted to examine the following models: the dysphoria model of PTSD, four-factor rumination model, and combined dysphoria and four-factor rumination model. Error covariances were fixed to zero, and factor variances were fixed to 1 to standardize factors in each model. Goodness of fit indices (in addition to the chi-square statistic, which has limitations) [25] that are reported below are the following: comparative fit index (CFI), Tucker Lewis Index (TLI), and root mean square error of approximation (RMSEA). According to Hu and Bentler [26], models with good fit (adequate fit statistics are in parentheses) have the following fit statistics: CFI and TLI ≥ .95 (.90-.94) and RMSEA ≤ .06 (.07-.08).

The first CFA conducted was on the dysphoria model of PTSD. PSS items were treated as ordinal, as research has shown this is the appropriate way to treat data with fewer than five response options [27, 28]. To examine the dysphoria model of PTSD, we specified the following factor loadings for the PSS items: items 1–5 on the PTSD-RE factor, items 6–7 on the PTSD-AV factor, items 8–15 on the PTSD-DYS factor, and items 16–17 on the PTSD-HYP factor. We used weighted least squares with a mean- and variance-adjusted (WLSMV) chi-square for CFA, which is the preferred estimator for ordinal/categorical items [27, 28]. Next, we conducted CFA on the 15 RTSQ items used to construct the four-factor rumination model [12]. Rumination items were treated as continuous, and we used maximum likelihood estimation with robust standard errors. To examine the four-factor rumination model, RTSQ items 1–4 were specified to load onto the R-RT factor, RTSQ items 5–8 on the R-CT factor, RTSQ items 9 and 11–14 on the R-PFT factor, and RTSQ items 17 and 20 on the R-AT factor. Following the recommendations of Tanner, Voon, Hasking and Martin [12], RTSQ items 10, 15–16, and 18–19 were not included in this model. Lastly, we examined the combined dysphoria and four-factor rumination model by using a WLSMV estimator and allowing all factors to correlate.

We tested the null hypothesis that the difference between two correlations would be zero by using Wald chi-square tests of parameter constraints. We examined which factors of rumination were most correlated with the PTSD-DYS factor and the PTSD-RE factor in the dysphoria model of PTSD.

## Results

Among the 304 trauma-exposed subjects, there were 109 men (35.9%). Age ranged from 19 to 64 years old (*M* = 42.56, *SD* = 11.66), and participants had between 7 to 19 years of education (*M* = 12.89, *SD* = 2.12). Most identified their race and ethnicity as Caucasian (*n* = 244, 80.3%), Native American (*n* = 30, 9.9%), and Hispanic (*n* = 28, 9.2%). Marital status was also recorded, with the following percentages: single (*n* = 153, 50.3%), married (*n* = 86, 28.3%), and previously married (*n* = 65, 21.4%). Most were employed part-time and full-time (*n* = 187, 61.5%), with the rest being either unemployed (*n* = 103, 33.9%), or retired (*n* = 14, 4.6%). Annual household income level was the following: < $15,000 (*n* = 128, 42.1%), $15,000–$25,000 (*n* = 82, 27.0%), $25,000–$35,000 (*n* = 48, 15.8%), $35,000-$50,000 (*n* = 26, 8.6%), and > $50,000 (*n* = 20, 6.6%).

Out of the 304 participants, 116 (38.2%) met *DSM-IV* criteria for a probable PTSD diagnosis. Total prevalence of probable PTSD in our sample of 411 participants was 28.2%. This is higher than the prevalence of PTSD in the general population [29]; however, since this is a primary care sample the prevalence of PTSD would be expected to be higher since there is a large relationship between both trauma and PTSD and utilization of healthcare [30, 31]. Total scores for the PSS in the remaining participants (*n* = 304) ranged from 0 to 47, with an average score of 12.17 (*SD* = 12.09), and the RTSQ had scores ranging from 23 to 140, averaging 77.59 (*SD* = 25.46).

CFA results indicated an adequately-fitting four-factor dysphoria model of PTSD, robust χ^2^(*df* = 113, *N* = 304) = 342.51, *p* < 0.001, CFI = 0.97, TLI = 0.97, RMSEA = 0.08 (see Table 2 and Table 3). The four-factor rumination model also had an adequate fit, Y—B χ^2^(*df* = 84, *N* = 304) = 251.55, *p* < 0.001, CFI = 0.92, TLI = 0.90, RMSEA = 0.08 (see Table 4 and Table 5). Lastly, the CFA for the combined dysphoria and four-factor RTSQ model indicated good fit, robust χ^2^(*df* = 436, *N* = 304) = 734.10, *p* < 0.001, CFI = 0.95, TLI = 0.95, RMSEA = 0.05 (see Table 6).

**Table 2:** Standardized Factor Loadings for the PTSD Dysphoria Model.

Items	Reexperiencing	Avoidance	Dysphoria	Hyperarousal
Intrusive thoughts	0.85			
Nightmares	0.77			
Reliving trauma	0.91			
Emotional cue reactivity	0.91			
Physiological cue reactivity	0.86			
Avoidance of thoughts		0.87		
Avoidance of reminder		0.87		
Trauma-related amnesia			0.55	
Loss of interest			0.86	
Feeling detached			0.88	
Feeling numb			0.83	
Hopelessness			0.73	
Difficulty sleeping			0.82	
Irritability/anger			0.82	
Difficulty concentrating			0.83	
Hypervigilance				0.88
Easily startled				0.92

*Note*. All factor loadings are significant at *p* < 0.001 level.

**Table 3:** Factor Correlations for the PTSD Dysphoria Model.

Factor Correlation	Reexperiencing	Avoidance	Dysphoria	Hyperarousal
Avoidance	0.90	1		
Dysphoria	0.89	0.92	1	
Hyperarousal	0.73	0.87	0.92	1

*Note*. All factor correlations are significant at *p* < 0.001 level.

**Table 4:** Standardized Factor Loadings for the Four-Factor Rumination Model.

Items	Rumination Factors			
	Problem-Focused Thoughts	Counterfactual Thinking	Repetitive Thinking	Anticipatory Thinking
When trying to solve a complicated problem, I find that I just keep coming back to the beginning, without ever finding a solution	0.70			
I have never been able to distract myself from unwanted thoughts	0.74			
Even if I think about a problem for hours, I still have a hard time coming to a clear understanding	0.92			
It is very difficult for me to come to a clear conclusion about some problems, no matter how much I think about it	0.86			
Sometimes I realise I have been sitting and thinking about something for hours	0.67			
When I am expecting to meet someone, I will imagine every possible scenario and conversation		0.50		
I tend to replay past events as I would have liked them to happen		0.73		
I find myself daydreaming about things I wish I had done		0.80		
When I feel I have had a bad interaction with someone, I tend to imagine various scenarios where I would have acted differently		0.74		
I find that my mind goes over things again and again			0.81	
When I have a problem, it will gnaw on my mind for a long time			0.83	
I find that some thoughts come to my mind over and over throughout the day			0.88	
I can’t stop thinking about some things			0.79	
When I am looking forward to an exciting event, thoughts of it interfere with what I am working on				0.68
If I have an important event coming up, I can’t stop thinking about it				0.79

*Note*. All factor loadings are significant at *p* < 0.001 level.

**Table 5:** Factor Correlations for the Four-Factor Rumination Model.

Factor Correlation	Problem-Focused Thinking	Counterfactual Thinking	Repetitive Thinking	Anticipatory Thinking
Counterfactual Thinking	0.71	1		
Repetitive Thinking	0.62	0.67	1	
Anticipatory Thinking	0.91	0.76	0.71	1

*Note*. All factor correlations are significant at *p* < 0.001 level.

**Table 6:** Factor Correlations for the Combined Dysphoria Model of PTSD and Four-Factor Rumination Model.

Factor Correlation	Problem-Focused Thinking	Counterfactual Thinking	Repetitive Thinking	Anticipatory Thinking
Reexperiencing	0.41	0.30	0.31	0.43
Dysphoria	0.49	0.35	0.40	0.49
Avoidance	0.38	0.40	0.39	0.43
Hyperarousal	0.42	0.46	0.44	0.46

*Note*. All factor correlations are significant at *p* < 0.001 level.

We conducted Wald chi-square tests of parameter constraints to examine differential relations between the PTSD-DYS factor and different factors of rumination (see Table 7). Results yielded two significant findings, which showed that the PTSD-DYS factor related more to both R-PFT (*r* = 0.49), Wald χ^2^(1, *N* = 304) = 8.50, *p* = 0.00, and R-AT (*r* = 0.49), Wald χ^2^(1, *N* = 304) = 6.82, *p* = 0.01, than R-CT (*r* = 0.35). No difference was found for dysphoria’s relationship to R-PFT and R-RT (*r* = 0.40), Wald χ^2^(1, *N* = 304) = 2.76 *p* = 0.10, or R-PFT and R-AT, Wald χ^2^(1, *N* = 304) = 0.01, *p* = 0.94. There were also no differences for the PTSD-DYS factor between R-RT and R-CT, Wald χ^2^(1, *N* = 304) = 0.89, *p* = 0.34, and R-RT and R-AT, Wald χ^2^(1, *N* = 304) = 2.59, *p* = 0.11.

**Table 7:** Correlations between the Four-Factor Rumination Model and PTSD’s Dysphoria Factor, and the Corresponding Wald Test Values.

Path	*r* (p-value)	Path	*r* (p-value)	*Wald χ2* (p-value)
PFT with DYS	0.49 (0.000)	CT with DYS	0.35 (0.000)	8.14 (.00)**
PFT with DYS	0.49 (0.000)	RT with DYS	0.40 (0.000)	2.76 (0.10)
PFT with DYS	0.49 (0.000)	AT with DYS	0.49 (0.000)	0.01 (0.94)
CT with DYS	0.35 (0.000)	RT with DYS	0.40 (0.000)	0.89 (0.34)
CT with DYS	0.35 (0.000)	AT with DYS	0.49 (0.000)	5.96 (.01)*
RT with DYS	0.40 (0.000)	AT with DYS	0.49 (0.000)	2.59 (0.11)
PFT with RE	0.41 (0.000)	CT with RE	0.30 (0.000)	4.42 (0.04) *
PFT with RE	0.41 (0.000)	RT with RE	0.31 (0.000)	3.60 (0.06)
PFT with RE	0.41 (0.000)	AT with RE	0.43 (0.000)	0.20 (0.66)
CT with RE	0.30 (0.000)	RT with RE	0.31 (0.000)	0.00 (0.98)
CT with RE	0.30 (0.000)	AT with RE	0.43 (0.000)	3.98 (0.05) *
RT with RE	0.31 (0.000)	AT with RE	0.43 (0.000)	4.17 (0.04) *

*Note*. PFT = Problem-Focused Thinking from RTSQ. CT = Counterfactual Thinking from RTSQ. RT = Repetitive Thinking from RTSQ. AT = Anticipatory Thinking from RTSQ.

RE = PTSD’s Reexperiencing DYS = PTSD’s Dysphoria.

*p < 0.05.

**p < 0.01.

We then conducted Wald chi-square tests of parameter constraints to examine the relationship between the PTSD-RE factor and the four factors of rumination (see Table 7). There were three significant findings, specifically showing that R-AT (*r* = 0.43) related more to the PTSD-RE factor than R-RT (*r* = 0.31), Wald χ^2^(1, *N* = 304) = 4.17, *p* = 0.04, and R-CT (*r* = 0.30), Wald χ^2^(1, *N* = 304) = 3.98, *p* = 0.05. Additionally, R-PFT (*r* = 0.41) was more related to PTSD-RE than R-CT (*r* = 0.30), Wald χ^2^(1, *N* = 304) = 4.42, *p* = 0.04. No significant differences for PTSD-RE were found between R-RT and R-CT, Wald χ^2^(1, *N* = 304) = 0.00, *p* = 0.98, R-RT and R-PFT, Wald χ^2^(1, *N* = 304) = 3.60, *p* = 0.06, and R-PFT and R-AT, Wald χ^2^(1, *N* = 304) = 0.20, *p* = 0.66.

## Discussion

We evaluated the relationship between rumination and the PTSD-DYS factor, and between rumination and the PTSD-RE factor, using the four-factor rumination model and the dysphoria model of PTSD. Because of R-PFT’s relationship to faulty cognitive processing [12] and similarities to the faulty processing seen as the result of rumination in the Ehlers and Clark [3] cognitive model of PTSD, we hypothesized it would be the most related to PTSD-DYS and PTSD-RE.

### Main Findings

Consistent with past research we found overall good fit for the dysphoria model [15]. We also found a good fit for the four-factor rumination model derived from 15 items from the RTSQ [12]. This confirms the four-factor model revealed by Tanner, Voon, Hasking and Martin [12]. This finding also provides additional support for rumination as a multi-dimensional construct [12], in contrast to the unidimensional model originally proposed by Brinker and Dozois [11]. Lastly, we found a good fit for a combined dysphoria and rumination model. When examining factor-level relations, we partially confirmed our initial hypothesis that R-PFT would more relate to the PTSD-DYS and PTSD-RE factors over other rumination factors. Specifically, the PTSD-DYS and PTSD-RE factor related more to R-PFT and R-AT than to R-CT. An additional finding was that the PTSD-RE factor related more to R-AT than to R-RT.

### Implications of Findings and Areas of Future Research

Results of the current study have several theoretical, research and clinical implications. First, the R-PFT factor is best conceptualized as deficits in cognitive information processing and problem-solving, and appears to be theoretically similar to the conceptualization of rumination put forth by Nolen-Hoeksema [32] and the Rumination Sadness Scale [33]. Tanner, Voon, Hasking and Martin [12] found that the problem-solving factor increased risk of psychological distress. On the other hand, R-CT represents a way of thinking in which a person considers alternative scenarios and imagines the alternative consequences. This can also be thought of as a “what-if” style of thinking [12]. No relation was found between this factor and psychological distress [12]. Therefore, because the PTSD-DYS factor is related to general emotional distress, the outcome of dysphoria being more related to problem-focused thinking than counterfactual thinking is not surprising. Additionally, the relationship between R-PFT and PTSD-RE appears to be clear because the faulty processing seen in R-PFT is consistent with the faulty processing posited by Ehlers and Clark [3].

Second, the finding that the PTSD-DYS and PTSD-RE factor was more related to R-AT than R-CT is interesting. In the Tanner, Voon, Hasking and Martin [12] study, they found that R-AT appeared to have a protective factor against psychological distress; however, it was unknown if this was due to future-oriented rumination that was positive in nature. Indeed, the RTSQ appears to be ambiguous as to the nature of future-oriented thinking, so participants in our sample could have been experiencing negative future-oriented thinking, as compared to those in the Tanner, Voon, Hasking and Martin [12] study. Considering that, in the context of PTSD, people tend to ruminate about the causes and consequences of a traumatic event [6], future-oriented thinking about the upcoming consequences of a traumatic event would result in negative affect [7] due to faulty processing of the traumatic memory. It could be due to this faulty processing and resulting negative affect that rumination’s anticipatory thinking factor was more related to PTSD-DYS. When it comes to R-AT’s relation to PTSD-RE, if participants in our sample were experiencing future-oriented ruminative thinking that was focused on consequences of the trauma, this would have served as a constant reminder of the trauma itself, possibly resulting in heightened reexperiencing symptoms.

Lastly, the additional finding that R-AT is more related than R-RT to PTSD-RE must be considered. Since the intrusive images that are part of the PTSD-RE factor are not processed efficiently, this would result in more intrusive images and PTSD symptoms [7]. Although, intuitively, one would then expect PTSD-RE to be more related to R-RT due to repeated intrusive images, the R-RT factor places more of an emphasis on the repetitive images, not the content. It could be that the *content* of future ruminative thoughts serve as constant reminders that the traumatic event happened, which could lead to heightened re-experiencing symptoms. This could account for PTSD-RE’s stronger co-relation with R-AT over R-RT.

The lack of other significant results is unexpected but suggests that only specific aspects of rumination are related to PTSD. For example, perhaps counterfactual thinking could serve as a protective factor because the person is imagining alternative scenarios that are more positive in nature, leading to altered perceptions of the traumatic memory and/or more tempered PTSD symptoms. Results also reflect the clinical importance of taking rumination into account when treating victims of trauma exposure, particularly those experiencing PTSD symptoms. Specifically, results may suggest the importance of working on problem-solving skills with victims that have been diagnosed with PTSD since their abilities to effectively process information could be compromised. Additionally, the results suggest that understanding the way people with PTSD are thinking about the future (particularly future consequences of the trauma) could impact their ability to process the actual events of the trauma and resulting negative mood. Skills utilized in trauma-focused therapy, such as cognitive restructuring about the causes and consequences of traumatic event exposure [34] could focus on teaching clients the actual realities of these consequences and working on their distorted beliefs surrounding the anticipated consequences; additionally, focusing on teaching clients basic problem-solving skills can go a long way in improving information processing of the traumatic event. Overall, the results indicate the importance of identifying content of rumination and being able to address it within a therapy context.

As this is a budding area of research, there are numerous avenues of research to be pursued. First, future studies should consider comparing other PTSD factors to other factors of rumination beyond the PTSD-DYS and PTSD-RE factor. There could be facets of rumination that simply are not accounted for by the distress symptoms in the PTSD-DYS factor, and relate more to other symptoms of PTSD beyond the intrusive thoughts aspect of the PTSD-RE factor. Furthermore, future studies should utilize *DSM-5* data to compare PTSD factors to rumination factors. *DSM-5* has four PTSD factors [35] based on the emotional numbing model [14]. The current study utilized *DSM-IV* data, so future studies should utilize data that reflect the most up to date factor structure for PTSD. Finally, evaluating the malleability of rumination factors in relation to specific PTSD factors is important to further refine treatment approaches to PTSD.

### Strengths

There are several strengths to this study that should be noted. First, this is one of the first studies to examine rumination’s relations to PTSD at the factor level, using the updated four factor models of both PTSD and rumination. Additionally, it also uses a more global measure of ruminative thinking, which does not confound the results with comorbid symptoms of depression and sadness, which could be the case with other questionnaires that measure rumination. Lastly, Tanner, Voon, Hasking and Martin [12] investigated the factor structure of the RTSQ in an adolescent sample. In this study, we extended these results by validating the factor structure they found in an adult sample.

### Limitations

There are also limitations to this study that should be acknowledged. First is the use of self-reported symptomatology. Second, although the RTSQ is a more global measure of ruminative thinking, there are other measures that measure rumination in more specific circumstances (e.g., rumination when depressed). There are possibly other measures of rumination that could be utilized in future studies to understand rumination’s relationship to PTSD in specific contexts. Third, this study only compared the PTSD-RE and PTSD-DYS factors and rumination factors. Therefore, the current study does not investigate the full scope of PTSD’s relationship with factors of rumination. Lastly, due to problems of shared method variance that can inflate variables’ relationships, it would be prudent for future work to measure relationships between PTSD factors and rumination factors using a mixed methods approach.

Overall, this study adds to the limited literature addressing rumination’s relationship with PTSD. This study is novel in that it compares both constructs at the latent factor level, using a newer factor model of rumination. As this is a relatively nascent area of research, this study provides a strong foundation to address the limitations and pursue future areas of research investigating cognitive vulnerabilities to PTSD and PTSD factor structure.

## Supporting Information

S1 Key PTSD and Rumination Key, with questionnaires for variables included in this study. (DOC)

S1 Data PTSD and Rumination Dataset, with data for the participants in this study. (CSV)

## References

[1] Elwood LS, Hahn KS, Olatunji BO, Williams NL. Cognitive vulnerabilities to the development of PTSD: a review of four vulnerabilities and the proposal of an integrative vulnerability model. Clin Psychol Rev. 2009;29: 87–100. 10.1016/j.cpr.2008.10.002 19008028

[2] Nolen-Hoeksema S, Morrow J, Fredrickson BL. Response styles and duration of episodes of depressed mood. J Abnorm Psychol. 1993;102: 20–28. 10.1037/0021-843X.102.1.20 8436695

[3] Ehlers A, Clark DM. A cognitive model of posttraumatic stress disorder. Behav Res Ther. 2000;38: 319–345. 10.1016/S0005-7967(99)00123-0 10761279

[4] Brewin CR, Holmes EA. Psychology theories of posttraumatic stress disorder. Clin Psychol Rev. 2003;23: 339–376. 10.1016/S0272-7358(03)00033-3 12729677

[5] Michael T, Ehlers A, Halligan SL, Clark DM. Unwanted memories of assault: what intrusion characteristics are associated with PTSD?. Behav Res Ther. 2005;43: 613–628. 10.1016/j.brat.2004.04.006 15865916

[6] Ehlers A, Steil R. Maintenance of intrusive memories in posttraumatic stress disorder: A cognitive approach. Behav Cogn Psychother. 1995;23: 217–249. 10.1017/S135246580001585X 21241539 PMC2887304

[7] Ehlers A, Mayou RA, Bryant R. Psychological predictors of chronic posttraumatic stress disorder after motor vehicle accidents. J Abnorm Psychol. 1998;167: 508–519. 10.1037/0021-843X.107.3.508 9715585

[8] Ehring T, Frank S, Ehlers A. The role of rumination and reduced concreteness in the maintenance of posttraumatic stress disorder and depression following trauma. Cognit Ther Res. 2008;32: 488–506. 10.1007/s10608-006-9089-7 20694036 PMC2908437

[9] Michael T, Halligan SL, Clark DM, Ehlers A. Rumination in posttraumatic stress disorder. Depress Anxiety. 2007;24: 307–317. 10.1002/da.20228 17041914

[10] Smith JM, Alloy LB. A roadmap to rumination: a review of the definition, assessment, and conceptualization of this multifaceted construct. Clin Psychol Rev. 2009;29: 116–128. 10.1016/j.cpr.2008.10.003 19128864 PMC2832862

[11] Brinker JK, Dozois DJ. Ruminative thought style and depressed mood. J Clin Psychol. 2009;65: 1–19. 10.1002/jclp.20542 19048597

[12] Tanner A, Voon D, Hasking P, Martin G. Underlying structure of ruminative thinking: Factor analysis of the ruminative thought style questionnaire. Cognit Ther Res. 2012;37: 633–646. 10.1007/s10608-012-9492-1

[13] Elhai JD, Palmieri PA. The factor structure of posttraumatic stress disorder: A literature update, critique of methodology, and agenda for future research. J Anxiety Disord. 2011;25: 849–854. 10.1016/j.janxdis.2011.04.007 21793239

[14] King DW, Leskin GA, King LA, Weathers FW. Confirmatory factor analysis of the clinician-administered PTSD scale: Evidence for the dimensionality of posttraumatic stress disorder. Psychol Assess. 1998;10: 90–96. 10.1037/1040-3590.10.2.90

[15] Simms LJ, Watson D, Doebbelling BN. Confirmatory factor analyses of posttraumatic stress symptoms in deployed and nondeployed veterans of the Gulf War. J Abnorm Psychol. 2002;111: 637–647. 10.1037//0021-843x.111.4.637 12428777

[16] Yufik T, Simms LJ. A meta-analytic investigation of the structure of posttraumatic stress disorder symptoms. J Abnorm Psychol. 2010;119: 764–776. 10.1037/a0020981 21090877 PMC4229035

[17] Elhai JD, Biehn TL, Armour C, Klopper JJ, Frueh BC, Palmieri PA. Evidence for a unique PTSD construct represented by PTSD’s D1–D3 symptoms. J Anxiety Disord. 2011;25: 340–345. 10.1016/j.janxdis.2010.10.007 21094021

[18] Clohessy S, Ehlers A. PTSD symptoms, response to intrusive memories and coping in ambulance service workers. Br J Clin Psychol. 1999;38: 251–265. 10.1348/014466599162836 10532147

[19] Forbes D, Parslow R, Creamer M, O’Donnell M, Bryant R, McFarlane A, et al A longitudinal analysis of posttraumatic stress disorder symptoms and their relationship with Fear and Anxious-Misery disorders: implications for DSM-V. J Affect Disord. 2010;127: 147–152. 10.1016/j.jad.2010.05.005 20605220

[20] Gros DF, Simms LJ, Acierno R. Specificity of posttraumatic stress disorder symptoms: An investigation of comorbidity between posttraumatic stress disorder symptoms and depression in treatment-seeking veterans. J Nerv Ment Dis. 2010;198: 885–890. 10.1097/NMD.0b013e3181fe7410 21135640

[21] Elhai JD, Naifeh JA, Forbes D, Ractliffe KC, Tamburrino M. Heterogeneity in clinical presentations of posttraumatic stress disorder among medical patients: testing factor structure variation using factor mixture modeling. J Trauma Stress. 2011;24: 435–443. 10.1002/jts.20653 21834086

[22] Goodman LA, Corcoran C, Turner K, Yuan N, Green BL. Assessing traumatic event exposure: General issues and preliminary findings for the stressful life events screening questionnaire. J Trauma Stress. 1998;11: 521–542. 10.1023/A:1024456713321 9690191

[23] Foa EB, Riggs DS, Dancu CV, Rothbaum BO. Reliability and validity of a brief instrument for assessing post-traumatic stress disorder. J Trauma Stress. 1993;6: 459–473. 10.1002/jts.2490060405

[24] Muthén BO, Muthén LK. Mplus (Version 6). Los Angeles, CA: Author; 2010.

[25] Kline RB. Principles and practice of structural equation modeling. 3rd edition New York: The Guilford Press; 2011.

[26] Hu L, Bentler PM. Cutoff criteria for fit indexes in covariance structure analysis: Conventional criteria versus new alternatives. Struct Equ Modeling. 1999;6: 1–55. 10.1080/10705519909540118

[27] Flora DB, Curran PJ. An empirical evaluation of alternative methods of estimation for confirmatory factor analysis with ordinal data. Psychol Methods. 2004;9: 466–491. 10.1037/1082-989X.9.4.466.supp 15598100 PMC3153362

[28] Wirth RJ, Edwards MC. Item factor analysis: Current approaches and future directions. Psychol Methods. 2007;12: 58–79. 10.1037/1082-989X.12.1.58.supp 17402812 PMC3162326

[29] Kessler RC, Sonnega A, Bromet E, Hughes M, Nelson CB. Posttraumatic stress disorder in the National Comorbidity survey. Arch Gen Psychiatry. 1995;52: 1048–1060. 10.1001/archpsyc.1995.03950240066012 7492257

[30] Elhai JD, North TC, Frueh BC. Health service use predictors among trauma survivors: A critical review. Psychol Serv. 2005;2: 3–19. 10.1037/1541-1559.2.1.3

[31] Pacella ML, Hruska B, Delahanty D. The physical health consequences of PTSD and PTSD symptoms: A meta-analytic review. J Anxiety Disord. 2013;27: 33–46. 10.1016/j.janxdis.2012.08.004 23247200

[32] Nolen-Hoeksema S. Responses to depression and their effects on the duration of depressive episodes. J Abnorm Psychol. 1991;100: 569–582. 10.1037/0021-843X.100.4.569 1757671

[33] Conway M, Csank PAR, Holm SL, Blake CK. On assessing individual differences in rumination on sadness. J Pers Assess. 2000;75: 404–425. 10.1207/S15327752JPA7503_04 11117154

[34] Barlow DH. Clinical handbook of psychological disorders: A step-by-step treatment manual. 4th edition: New York, NY: The Guilford Press; 2008 10.1007/s10597-009-9223-6

[35] American Psychological Association. Diagnostic and statistical manual of mental disorders. 5th ed Arlington, VA: American Psychiatric Publishing; 2013.

