# Supplementary material for: Assessing Relations between PTSD’s Dysphoria and Reexperiencing Factors and Dimensions of Rumination
Source: PLoS One. 2015 Mar 4;10(3):e0118435. doi: 10.1371/journal.pone.0118435 (PMC4349788; doi:10.1371/journal.pone.0118435)
Supplement: S1 Key — (DOC) [file pone.0118435.s001.doc]

**Please answer the questions as honestly as possible. Do your best to answer questions fully;**

**but you do not have to answer any question you prefer not to answer.**

1. What is your gender?  Male _(1)_  Female _(2)_

2. What is your current age? _______

3. How many years of schooling have you completed, *starting with first grade*? _____

(e.g., graduated high school = 12; graduated college = 16)

4. What is your current employment status? (check only one)  Part time _(1)_

Full time _(2)_

Retired _(3)_

Unemployed _(4)_

Unemployed Student _(5)_

5. For the majority of the past 12 months, what has your relationship status been? (check only one)

Single _(1)_

Living with Significant Other _(2)_

Married _(3)_

Divorced, Separated, or Widowed _(4)_

6. What was your current annual household income over the past year? (Check only one)

Less than $15,000 _(1)_  $35,000 to $49,999 _(4)_   $80,000 or higher _(7)_  $15,000 to $24,999 _(2)_  $50,000 to $64,999 _(5)_

$25,000 to $34,999 _(3)_  $65,000 to $79,999 _(6)_

**RUMINATIVE THOUGHT STYLE QUESTIONNAIRE**

Using the scale below, please rate each item in terms of how well it describes you

(1 = “Does not describe me at all”, 7 = “Describes me very well”). Please circle your answer, beside each question.

**Not at Very**

**All Well**

1. I find that my mind often goes over things again and again. 1 2 3 4 5 6 7

1. When I have a problem, it will gnaw on my mind for a long time. 1 2 3 4 5 6 7
2. I find that some thoughts come to mind over and over throughout the day. 1 2 3 4 5 6 7
3. I can’t stop thinking about some things. 1 2 3 4 5 6 7
4. When I am anticipating an interaction, I will imagine every possible scenario

and conversation. 1 2 3 4 5 6 7

1. I tend to replay past events as I would have liked them to happen. 1 2 3 4 5 6 7
2. I find myself daydreaming about things I wish I had done. 1 2 3 4 5 6 7
3. When I feel I have had a bad interaction with someone, I tend to imagine various

scenarios where I would have acted differently. 1 2 3 4 5 6 7

1. When trying to solve a complicated problem I find that I just keep coming

back to the beginning without ever finding a solution. 1 2 3 4 5 6 7

1. If there is an important event coming up, I think about it so much that I work

myself up. 1 2 3 4 5 6 7

1. I have never been able to distract myself from unwanted thoughts. 1 2 3 4 5 6 7
2. Even if I thought about a problem for hours, I still have a hard time

coming to a clear understanding. 1 2 3 4 5 6 7

13. It is very difficult for me to come to a clear conclusion about some problems,

no matter how much I think about it. 1 2 3 4 5 6 7

14. Sometimes I realize I have been sitting and thinking about something for hours. 1 2 3 4 5 6 7

15. When I am trying to work out a problem, it is like I have a long debate in my

mind where I keep going over different points. 1 2 3 4 5 6 7

16. I like to sit and reminisce about pleasant events from the past. 1 2 3 4 5 6 7

17. When I am looking forward to an exciting event, thoughts of it interfere with

what I am working on. 1 2 3 4 5 6 7

18. Sometimes even during a conversation, I find unrelated thoughts popping into

my head. 1 2 3 4 5 6 7

19. When I have an important conversation coming up I tend to go over it in my

mind again and again. 1 2 3 4 5 6 7

20. If I have an important event coming up, I can’t stop thinking about it. 1 2 3 4 5 6 7

**STRESSFUL LIFE EVENTS SCREENING QUESTIONNAIRE**

The following questions ask about many different types of stressful life events.

These kinds of events can be frightening or distressing to almost everyone.

During your life, have any of the following events ever happened to you?

*(For each question, please check the answer choice that applies to you)*

1. Have you ever had a life-threatening illness?

Yes____1 No____2

2. Were you ever in a life-threatening accident?

Yes____1 No____2

3. Was physical force or a weapon ever used against you in a robbery or mugging?

Yes____1 No____2

4. Has an immediate family member, romantic partner or VERY CLOSE friend died as a result

of accident, homicide, or suicide?

Yes____1 No____2

5. When you were a child or more recently, did anyone (parent, other family member, romantic partner,

stranger or someone else) ever succeed in PHYSICALLY FORCING you to have intercourse, or oral

or anal sex against your wishes OR when you were in some way helpless?

Yes____1 No____2

6. Other than experiences described in the previous item, has anyone ever used PHYSICAL FORCE

OR THREAT to TRY to make you have intercourse, oral or anal sex, against your wishes OR when

you were in some way helpless?

Yes____1 No____2

7. Other than experiences mentioned in the previous two items, has anyone ever ACTUALLY

TOUCHED private parts of your body or made you touch theirs against your wishes, OR when you

were in some way helpless?

Yes____1 No____2

8. When you were a child, did a parent, caregiver or other person ever slap you repeatedly, beat or

otherwise attack or harm you?

Yes____1 No____2

9. Other than the experiences mentioned in the previous item, have you ever been kicked, beaten,

slapped around or otherwise physically harmed by a romantic partner, date, sibling, family member,

stranger or someone else?

Yes____1 No____2

10. Other than the experiences already covered, has anyone ever THREATENED you with a weapon,

like a knife or gun?

Yes____1 No____2

11. Have you ever been present when another person was killed, seriously injured, or sexually or

physically assaulted?

Yes____1 No____2

12. Have you ever been in any other situation where you were seriously injured or your life was in danger

(e.g., involved in military combat or living in a war zone)?

Yes____1 No____2

Please describe.____________________________________________________________________________

13. Have you ever been in any other situation that was extremely frightening or horrifying

that has not been covered above?

Yes____1 No____2

Please describe.____________________________________________________________________________

14. The events listed below correspond to the 13 items queried above. If any of these events happened to you,

CIRCLE the *ONE* event (only 1) that CAUSES YOU THE MOST DISTRESS (even if it doesn’t really cause

you much distress). If none of these events happened to you, check “None” at the bottom of this list.

**_______________________________________________________________________________________**

1. Life-threatening illness 8. Parent/caregiver physically harmed you

2. Life-threatening accident 9. Partner/date etc. physically harmed you

3. Physical force/weapon used 10. Threatened with a weapon

4. Family member/close friend died 11. Present when someone was killed, injured, or assaulted

5. Physical force used to have sex 12. Other situation: seriously injured/life in danger

6. Physical force/threat to try to have sex 13. Other situation: extremely frightening/horrifying

7. Childhood: Touched your body private parts 14. None of these events happened to me

When did this event (first) occur? (your age): ____________________

(a)When did this event last occur? (your age): ____________________

(b) Did you experience intense fear, helplessness or horror when it happened? yes_1_  / no_2_

(c) **How much distress** (anxiety, worry, sadness, frustration, or grief) **does this event cause you?**

(*Circle the best answer*)

None happened No Slight Moderate Considerable Extreme

To me_1_ Distress_2_ Distress_3_ Distress_4_ Distress_5_ Distress

**PTSD SYMPTOM SCALE**

Using the scale below, please rate each item in terms of how frequent/severe it has been for you,

in the past two weeks, *based on the most distressing event you reported* from the previous page.

(If you did not experience any events listed on the previous page, please skip to the next page).

Please circle your answer, beside each question.

SCALE

**0 = Not at all**

**1 = Once per week or less/a little bit/once in a while**

**2 = 2 to 4 times per week/somewhat/half the time**

**3 = 5 or more times per week/very much/almost always**

**__________________________________________________________________________________________**

1. Have you had recurrent or intrusive distressing thoughts or recollections

about the trauma? 0 1 2 3

2. Have you been having recurrent bad dreams about the trauma? 0 1 2 3

3. Have you had the experience of suddenly reliving the trauma, flashbacks of it,

acting or feeling as if it were re-occurring? 0 1 2 3

4. Have you been intensely emotionally upset when reminded of the trauma? 0 1 2 3

5. Have you been having intense physical reactions when reminded of the trauma? 0 1 2 3

6. Have you persistently been making efforts to avoid thoughts or feelings associated

with the trauma? 0 1 2 3

7. Have you persistently been making efforts to avoid activities, situations or places

that remind you of the trauma? 0 1 2 3

8. Are there any important aspects of the trauma that you still cannot remember? 0 1 2 3

9. Have you markedly lost interest in free time activities since the trauma? 0 1 2 3

10. Have you felt detached or cut off from others around you since the trauma? 0 1 2 3

11. Have you felt that your ability to experience emotions is less? 0 1 2 3

12. Have you felt that any future plans or hopes have changed because of the trauma? 0 1 2 3

13. Have you been having persistent difficulty falling or staying asleep? 0 1 2 3

14. Have you been continuously irritable or having outbursts of anger? 0 1 2 3

15. Have you been having persistent difficulty concentrating? 0 1 2 3

16. Are you overly alert since the trauma? 0 1 2 3

17. Have you been jumpier, more easily startled, since the trauma? 0 1 2 3

**END OF SURVEY QUESTIONS**
